# Relationship between caesarean section and breastfeeding: evidence from the 2013 Turkey demographic and health survey

**DOI:** 10.1186/s12884-020-2732-6

**Published:** 2020-01-28

**Authors:** Nüket Paksoy Erbaydar, Tuğrul Erbaydar

**Affiliations:** 1 Faculty of Medicine, Department of Public Health, Hacettepe University, Sıhhiye, 06100 Ankara, Turkey; 2 Faculty of Medicine, Department of Public Health, Ankara University, Ankara, Turkey

**Keywords:** Caesarean section, Breastfeeding, Cohort study, Demographic and health survey

## Abstract

**Background:**

The mode of delivery influences breastfeeding practices. High rates of caesarean section and low breastfeeding rates are important public health concerns for all developing countries. This study aimed to determine the relationship between caesarean section and early breastfeeding practices among primiparae.

**Methods:**

Data for primiparae with a singleton birth (*N* = 777) obtained from the 2013 Turkey Demographic and Health Survey were used in this retrospective cohort study. Early initiation of breastfeeding within one hour of delivery and exclusive breastfeeding during the first three days following birth were evaluated. Standardised incidence rates and standardised rate ratios of non-early initiation of breastfeeding and non-exclusive breastfeeding were calculated according to the mode of delivery.

**Results:**

The late initiation of breastfeeding and non-exclusive breastfeeding incidence rates were 42.7 and 41.0%, respectively. The standardised incidence rate of late initiation of breastfeeding among women with vaginal delivery was 35.34%, versus 50.49% among those with caesarean delivery. The standardised rate ratios for late initiation of breastfeeding and non-exclusive breastfeeding were 1.428 (95% confidence interval (CI): 1.212–1.683) and 1.468 (95% CI: 1.236–1.762), respectively. Women who underwent caesarean section had a higher risk of late initiation of breastfeeding and non-exclusive breastfeeding during the three days following delivery, after controlling for sociodemographic and delivery-related factors.

**Conclusions:**

This study provides useful evidence for the implementation of strategies to prevent unnecessary caesarean sections, which negatively affect not only maternal health but also neonatal health. The promotion of mother-friendly policies by healthcare institutions, implemented in a baby-friendly manner, is essential.

## Background

Breastfeeding is essential to the health of infants and young children. Colostrum is defined as the ‘perfect food’ for newborns, and the World Health Organization (WHO) recommends that breastfeeding be initiated within one hour of delivery [1]. In 2012, the World Health Assembly (WHA) endorsed a plan to increase the rate of exclusive breastfeeding (EBF) during the six months after delivery to ≥50% by 2025 [2].

**Fig. 1:**
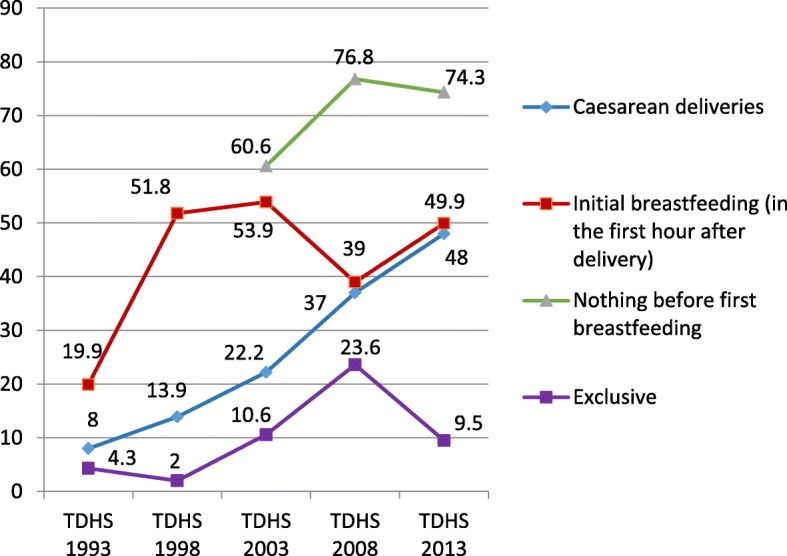
Breastfeeding trends and related indicators according to the Turkey Demographic and Health Surveys

Demographic and health surveys (DHSs) provide nationally representative population, reproduction, and child nutrition data in many countries, including Turkey. Trends for some indicators regarding breastfeeding in Turkey, according to the five most recent Turkey DHSs (TDHSs), are shown in Fig. 1. According to the 2013 TDHS, 49.9% of newborns received early breastfeeding and 74.3% of newborns did not receive any type of nutrition prior to breastfeeding [3]. In 57 low and middle-income countries in Asia, Latin America, the Middle East, Europe, and Sub-Saharan Africa where DHSs were performed between 2000 and 2013, the overall rate of early initiation of breastfeeding (EIBF) was 39% and that of avoidance of prelacteal feeding was 49.2% [4]. The rates of EIBF in WHO European Region member states varies widely; the prevalence of EIBF in the 1998–2012 period was 4.6% in Bulgaria, 33.5% in Ireland, 66.5% in Luxembourg, 78.1% in Austria, and 83.8% in Kyrgyzstan [5].

The Ministry of Health of Turkey has initiated multiple programmes to improve maternal and child health, and hospital deliveries gradually increased from 91.3% in 2008 to 98.0% in 2013. Further, there has been a gradual increase in the number of baby-friendly hospitals. Following the initiation of the ‘Baby-Friendly Health Institutions Programme’ in 1991, 53.2% of hospitals were certified as baby-friendly by 2008; this proportion increased to 72.2% in 2013 [6]. The increase in hospital deliveries and implementation of policies that support breastfeeding were expected to improve breastfeeding practices and breastfeeding-related indicators, but as seen in Fig. 1, breastfeeding indicators are inconsistent and far from the child nutrition targets of the 65th WHA [2]. This indicates the multifactorial nature of breastfeeding behaviour [7, 8] and makes it evident that programmes to improve breastfeeding practices need to be fine-tuned.

Mode of delivery is among the many factors that affect breastfeeding practices. In addition, studies reporting the negative consequences of caesarean section (C/S) on the well-being and behaviour of new mothers and the physiology of lactation during the early postpartum period continue to increase in number [9–13]. C/S is considered major abdominal surgery, and post-surgical procedures for mothers and routine procedures for newborns can delay EIBF. Within a few hours of C/S, new mothers are expected to begin caring for their newborns while simultaneously coping with the problems associated with the post-surgery period, including post-surgical pain [14], which can negatively affect EIBF. The quality of support that a new mother receives following C/S can help facilitate breastfeeding to a degree, but the negative effects of surgery and the physiological effects of C/S on lactation can persist.

According to 2013 TDHS data, the C/S rate in Turkey was 48% (Fig. 1) [3]. In 2017, this rate increased to 53.1%, and the primary C/S rate was 25.79% [15]. As C/S rates remain high in many countries including Turkey and lack of breastfeeding continues to be a major global public health concern, the relationship between C/S and breastfeeding requires further clarification. As such, the present study aimed to determine the attributable effect of C/S on breastfeeding using 2013 TDHS data, while controlling for the effects of sociodemographic and delivery-related factors.

## Methods

**Fig. 2:**
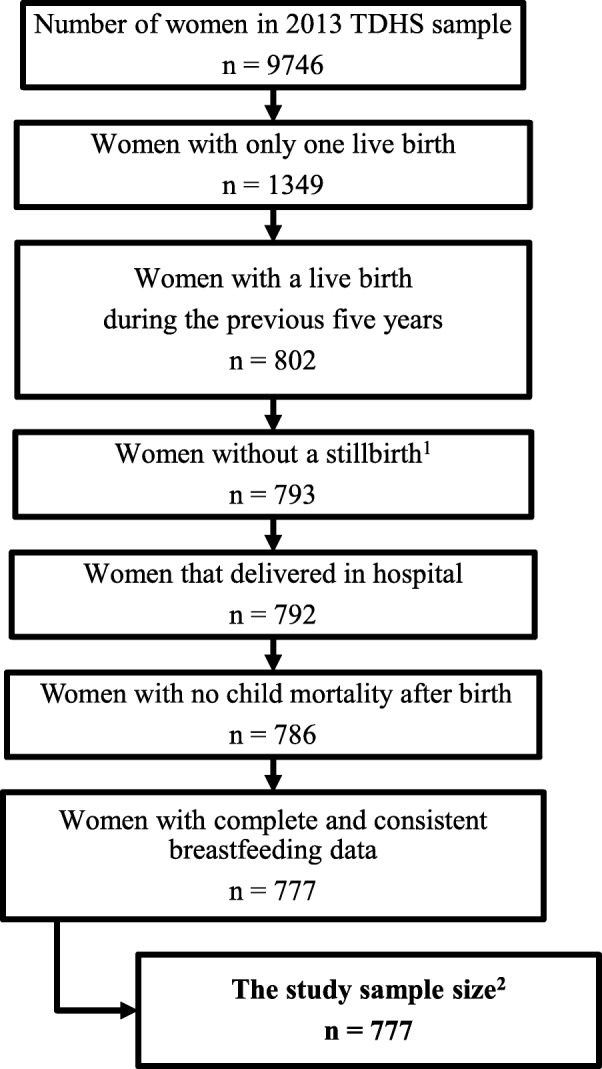
Flow diagram of study sampling. ^1^Women with a history of stillbirth were excluded from the study even if they had a live birth during the previous five years. ^2^The sample consisted of primiparae who had given birth in hospital during the previous five years, had not experienced stillbirth or the death of a child, and had complete and consistent breastfeeding data

Data for this study were culled from the 2013 TDHS, which used a weighted multistage, stratified cluster sampling approach. This sampling design aimed to ensure that the survey provided estimates with acceptable precision for all of Turkey. Interviewer-administered structured questionnaires were used to collect data for the survey. In the 2013 TDHS, the women’s questionnaire, designed for those aged between 15 and 59 years and listed in households, was used to collect data on maternal health characteristics, such as mode of delivery and breastfeeding experiences, between 2008 and 2013. In the present study, the 2013 TDHS data were analysed using a retrospective cohort design, so as to determine the relationship between mode of delivery and early breastfeeding practices. In total, 9746 women were interviewed for the 2013 TDHS. In order to eliminate any possible effects of previous birth experiences, only data for women who gave birth in hospital to their first and only live child within five years of the survey were included in the present study. Consequently, the final subset of data consisted of 777 women. A flow diagram of the study sampling is shown in Fig. 2.

According to breastfeeding indicators developed by the WHO [16] and used for the 2013 TDHS [3], EIBF (breastfeeding that begins within one hour of delivery) and EBF (feeding only with breast milk) for the first three days post-delivery were used to define the outcome variables. Accordingly, initiation of breastfeeding ≥1 h after delivery or no breastfeeding were classified as non-EIBF. Any nutritional intake in addition to breast milk or no breastfeeding in the first three days following birth was considered non-EBF.

The independent variables were grouped sociodemographic and delivery-related data. Sociodemographic data included maternal age, educational level (the three levels were none-some primary school, primary school-some secondary school, and secondary school-university), occupation, place of residence (rural or urban), residential region (Northern, Southern, Eastern, or Western Anatolia), and socioeconomic status. Socioeconomic status was determined using a wealth index of household assets and was grouped into quintiles (poorest, poor, middle, rich, richest). Delivery-related data included mode of delivery (vaginal delivery [VD] or C/S), place of delivery (public hospital or private hospital), and sex of the newborn. The chi-square test was used to evaluate the relationships between the independent and outcome variables. Binary logistic regression analysis was performed using data for the two main outcomes: non-EIBF and non-EBF.

Data were analysed using IBM SPSS Statistics for Windows v.23.0 (IBM Corp., Armonk, NY). The focus of statistical analysis was to determine if mode of delivery had any effect on breastfeeding practices; therefore, the incidence of non-EIBF and non-EBF in the VD and C/S groups were compared. Relative risks (RRs) were calculated as unadjusted measures of comparison, and then standardised incidence rates (SIRs) and standardised rate ratios (SRRs) were determined. Based on the results of the logistic regression analysis, mothers’ residential region and educational level were used as control variables.

The direct standardisation method was used. The non-EIBF and non-EBF incidence rates in the VD and C/S groups were calculated for each combination of mothers’ educational level and residential region. The entire study group was used as the standard population. Maternal educational level and region-specific incidence rates were projected to the standard population, and SIRs in the C/S and VD groups were calculated. Then, SRRs were calculated by dividing the SIRs in the C/S group by the SIRs in the VD group. Statistical significance for all analyses was set at *P* <  0.05 and 95% confidence intervals (CI) were obtained using bivariate and multiple logistic regression.

## Results

The mean age of the 777 primiparae was 26.3 ± 5.3 years. In 47.7% of the participants, the educational level was below secondary school, 17.4% were in the first wealth index quintile, 77.9% lived in urban areas, 26.4% were from the Eastern Anatolian region, and 60.5% were employed. In total, 58.9% of the deliveries occurred in public hospitals and 53.7% of all deliveries were via C/S. Among the newborns, 56.4% were boys. The frequency of non-EIBF and non-EBF was 42.7 and 41.0%, respectively (Table 1).

**Table 1:** Delivery and breastfeeding characteristics of primiparae

	Number	Percent
*Delivery-related characteristics*		
Place of delivery		
Public hospital	458	58.9
Private hospital	319	41.1
Mode of delivery		
VD^a^	360	46.3
C/S^b^	417	53.7
Sex of newborn		
Boy	438	56.4
Girl	339	43.6
*Breastfeeding practices*		
Within one hour after delivery		
EIBF^c^	445	57.3
Non-EIBF^d^	332	42.7
In the first three days after delivery (*N* = 776)		
EBF^e^	458	59.0
Non-EBF^f^	318	41.0

^a^Vaginal delivery

^b^Caesarean section

^c^Early initiation of breastfeeding

^d^Non-EIBF: Initiation of breastfeeding ≥ 1 h after delivery or no breastfeeding

^e^Exclusive breastfeeding in the first three days following birth

^f^Non-EBF: Any nutrition other than breast milk in the first three days following birth or no breastfeeding

In terms of education, 58.9% of the women with the lowest level (none-some primary school) were in the non-EIBF group, as compared to 41.2% of those with a primary school-some secondary school level and 40.8% of those with a secondary school-university level (*P* = 0.013). Conversely, 45.3% of the women with the highest educational level (secondary school-university) were in the non-EBF group, as compared to 38.0% of those with a primary school-some secondary school level and 36.1% of those with a none-some primary school level (*P* = 0.096). The relationship between place of residence and non-EBF status was significant; more women in the non-EBF group lived in urban areas (43.0%) (*P* = 0.033). There was no significant relationship between other sociodemographic variables (age, wealth index quintile, region, and occupation) and non-EIBF or non-EBF status (*P* > 0.05) (Table 2).

**Table 2:** Bivariate analysis of sociodemographic and breastfeeding practices of primiparae

Sociodemographic characteristics		Breastfeeding practices				P
		Non-EIBF^a^ (*N* = 777)	Total	Non-EBF^b^ (*N* = 776)	Total	
Age group (years)	15–19	43.4	53	34.6	52	0.439^*^ 0.090^**^
	20–24	43.7	270	38.9	270	
	25–29	40.4	255	37.6	255	
	30–34	46.7	135	51.1	135	
	35–39	45.1	51	45.1	51	
	40–49	15.4	13	53.8	13	
Education	None-some primary school	58.9	73	36.1	72	0.013^*^ 0.096^**^
	Primary school-some secondary school	41.2	371	38.0	371	
	Secondary school-university	40.8	333	45.3	333	
Wealth index	Poorest	48.9	135	30.4	135	0.212^*^ 0.078^**^
	Poor	46.5	172	40.9	171	
	Middle	37.0	165	42.4	165	
	Rich	40.0	150	44.7	150	
	Richest	41.9	155	45.2	155	
Residence	Urban	41.5	605	43.0	605	0.190^*^ 0.033^**^
	Rural	47.1	172	33.9	171	
Region	West	37.9	190	42.6	190	0.058^*^ 0.560^**^
	South	38.4	99	36.4	99	
	Central	40.0	160	40.6	160	
	North	43.1	123	46.3	123	
	East	51.2	205	38.7	204	
Occupation	Working	41.9	470	42.1	470	0.571^*^ 0.420^**^
	Not working	44.0	307	39.2	306	

^a^Non-EIBF: Initiation of breastfeeding ≥ 1 h following birth or no breastfeeding

^b^Non-EBF: Any nutrition other than breast milk in the first three days following birth or no breastfeeding

^*^P value for non-EIBF

^**^P value for non-EBF

The women who underwent C/S were more likely to belong to the non-EIBF and non-EBF groups than those who underwent VD (48.4% vs. 36.1% [*P* <  0.001] and 48.4% vs. 32.3% [P <  0.001], respectively). We did not observe a significant relationship between place of delivery and sex of the newborn and non-EIBF or non-EBF status (Table 3). Logistic regression analysis showed that maternal educational level and residential region were related to non-EIBF status. The women with the lowest educational level and those who lived in Eastern Anatolia had the highest risk of non-EIBF. Mode of delivery was the only variable that was significantly related to both non-EIBF and non-EBF. The risk of non-EIBF and the risk of non-EBF was higher in women who had undergone C/S (OR = 2.07 95% CI: 1.50–2.87 [*P* <  0.001] and OR = 1.94 95% CI: 1.40–2.67 [P <  0.001], respectively). None of other variables was significantly related to non-EBF (Table 4).

**Table 3:** Breastfeeding practices according to delivery-related features of primiparae

Delivery-related features	Breastfeeding practices				P
	Non-EIBF^a^ (*n* = 777)	Total	Non-EBF^b^ (*n* = 776)	Total	
Mode of delivery					
VD^c^	36.1	360	32.3	359	< 0.001^*^ < 0.001^**^
C/S^d^	48.4	417	48.4	417	
Place of delivery					
Public hospital	43.4	458	40.0	457	0.626^*^ 0.526^**^
Private hospital	41.7	319	42.3	319	
Sex of newborn					
Boy	43.6	438	42.1	437	0.573^*^ 0.469^**^
Girl	41.6	339	39.5	339	

^a^Non-EIBF: Initiation of breastfeeding ≥ 1 h following birth or no breastfeeding

^b^Non-EBF: Any nutrition other than breast milk in the first three days following birth or no breastfeeding

^c^Vaginal delivery

^d^Caesarean section

^*^P value for non-EIBF

^**^P value for non-EBF

**Table 4:** Logistic regression analysis of breastfeeding, sociodemographic, and delivery-related characteristics of primiparae

	Non-EIBF^a^			Non-EBF^b^		
	OR^c^	95% CI^d^	P	OR	95% CI	P
*Sociodemographics*						
Age	0.96	0.74–1.44	0.548	1.08	0.93–1.25	0.331
Educational level						0.917
No education-some primary school	1	Reference		1	Reference	
Primary school-some secondary school	0.56	0.33–0.97	0.038	1.00	0.57–1.76	0.99
Secondary school and university	0.53	0.29–0.99	0.047	1.08	0.57–2.03	0.813
Wealth index			0.636			0.762
Poorest	1	Reference		1	Reference	
Poor	1.05	0.63–1.75	0.848	1.40	0.82–2.38	0.214
Middle	0.74	0.41–1.32	0.301	1.35	0.74–2.45	0.325
Rich	0.93	0.49–1.77	0.835	1.46	0.76–2.80	0.261
Richest	0.94	0.47–1.89	0.864	1.31	0.64–2.66	0.460
Place of residence						
Rural	1	Reference		1	Reference	
Urban	0.91	0.60–1.40	0.670	1.20	0.78–1.85	0.406
Residential region			0.145			0.712
West	1	Reference		1	Reference	
South	0.93	0.55–1.56	0.799	0.81	0.47–1.37	0.422
Central	1.07	0.67–1.70	0.782	1.00	0.63–1.58	0.993
North	1.24	0.77–2.01	0.379	1.19	0.74–1.92	0.469
East	1.62	1.03–2.57	0.038	1.09	0.69–1.73	0.707
Occupation						
Working	1	Reference		1	Reference	
Not working	1.03	0.74–1.44	0.864	1.07	0.76–1.50	0.703
*Delivery-related characteristics*						
Place of delivery						
Public hospital	1	Reference		1	Reference	
Private hospital	0.93	0.66–1.30	0.664	0.83	0.59–1.16	0.279
Mode of delivery						
VD^e^	1	Reference		1	Reference	
C/S^f^	2.07	1.50–2.87	< 0.001	1.94	1.40–2.67	< 0.001
Sex of newborn						
Girl	1	Reference		1	Reference	
Boy	1.12	0.83–1.50	0.472	1.16	0.86–1.56	0.339

^a^Non-EIBF: Initiation of breastfeeding ≥ 1 h following birth or no breastfeeding

^b^Non-EBF: Any nutrition other than breast milk in the first three days following birth or no breastfeeding

^c^Odds ratio

^d^Confidence interval

^e^Vaginal delivery

^f^Caesarean section

In order to determine the risk of non-EIBF and non-EBF associated with C/S, the crude and adjusted incidences in the C/S and VD groups were compared. The incidence of non-EIBF was higher in the C/S group (48.4%) than in the VD group (36.1%), and C/S significantly increased the risk of non-EIBF (RR: 1.341; 95% CI: 1.132–1.589). On the contrary, the incidence of non-EBF was 48.4% in the C/S group, versus 32.3% in the VD group (RR: 1.499; 95% CI: 1.253–1.794). Based on direct standardisation, the SRR for non-EIBF was 1.428 (95% CI: 1.212–1.683), versus 1.468 for non-EBF (95% CI: 1.236–1.762) (Table 5).

**Table 5:** Unadjusted relative risk, standardised incidence rate, and standardised relative ratio for non-EIBF and non-EBF according to mode of delivery

Breastfeeding practices	Unadjusted incidence rate (%)		Unadjusted RR for C/Ss 95% CI^d^	SIR^a^ (%) 95% CI		SRR^a^ 95% CI
	VD^b^	C/S^c^		VD	C/S	
Non-EIBF^e^	36.111	48.441	1.341 (1.132–1.589)	35.343 (30.307–0.379)	50.485 (45.659–5.310)	1.428 (1.212–1.683)
Non-EBF^f^	32.312	48.441	1.499 (1.253–1.794)	33.405 (28.332–8.478)	49.044 (44.082–54.007)	1.468 (1.233–1.748)

^a^Standardised rates and ratios were calculated by controlling for residential region and educational level. The entire sample (*n* = 777 for non-EIBF and n = 776 for non-EBF) was used as the reference population for standardisation

^b^Confidence interval

^c^Vaginal delivery

^d^Caesarean section

^e^Non-EIBF: Initiation of breastfeeding ≥ 1 h following birth or no breastfeeding

^f^Non-EBF: Any nutrition other than breast milk during the first three days following birth or no breastfeeding

## Discussion

The present findings show that after controlling for sociodemographic and delivery-related factors, the women who had undergone C/S had a 1.428-fold higher risk of non-EIBF and a 1.468-fold higher risk of non-EBF than those who had undergone VD (Table 5). In response to a growing body of evidence, scientists have stated, ‘Never before in the history of science has so much been known about the complex importance of breastfeeding for both mothers and children’ [17]. Mode of delivery is among the factors that play an important role in breastfeeding practices. C/S can negatively affect the physiology of lactation and cause adverse events that hinder maternal contact with the neonate, resulting in intolerable post-surgical maternal pain and an increase in the level of need for intensive care required by neonates, both of which can negatively affect breastfeeding [10, 14, 18–20]. The present study’s multivariate analysis indicates that maternal educational level, residential region, and mode of delivery are significantly related to non-EIBF and that mode of delivery has a significant relationship with non-EBF. The literature shows that maternal educational level is among the most significant determinants of breastfeeding behaviour [21]; however, findings related to the effect of maternal educational level on breastfeeding behaviour are inconsistent. Studies from Iran [22] and Bahrain [23] reported that as maternal educational level increases, the likelihood of breastfeeding decreases, whereas studies from Argentina [24] and Italy [25] show that there is a positive association between maternal educational level and the likelihood of breastfeeding. Based on the present findings, we think that, owing to their use of modern information resources (communication with healthcare professionals and access to scientific books and the internet), mothers with a high educational level were well aware of its benefits and, therefore, highly motivated to feed their newborns with colostrum. Further, they fully cooperated with healthcare personnel during hospitalisation, even though their intention toward EBF in the days following delivery did not continue in all cases. These results indicate that maternal educational level might be a potential confounder for non EIBF and non-EBF.

As per the results of the bivariate analysis, there existed a significant relationship between place of residence and non-EBF (43.0% of the women with non-EBF status lived in urban areas, versus 33.9% in rural areas [*P* = 0.033]). However, the relationship between place of residence and non-EIBF failed to achieve significance (Table 2). Based on DHSs, Adewuyi et al. [19] and Pandey et al. [26] reported that non-EIBF rates are lower in women from rural areas. The significance of the relationship between place of residence and non-EBF in the present study disappeared in multivariate analysis. As such, we think that place of residence alone did not have a significant effect on breastfeeding practices in women who delivered in hospitals. The non-EIBF rate (51.2%) was highest in women from Eastern Anatolia, which is the least developed region of Turkey, whereas it (37.9%) was lowest in women from Western Anatolia (the most developed region) (Table 2). The difference in odds ratios (ORs) between these two regions was significant according to regression analysis (Table 4), indicating that residential region could be another confounder for non-EIBF and non-EBF.

In the present study, the risk of non-EIBF and non-EBF was observed to be related to C/S. The relative risk of non-EIBF was 1.341 (95% CI: 1.132–1.589) when the C/S and VD groups were compared without adjustments. After controlling for maternal educational level and residential region, the SIR was 1.428 based on the adjusted incidence rates for non-EIBF, which indicates that the risk of non-EIBF in women who had C/S was 1.428-fold higher (95% CI, 1.212–1.683) than in those with VD. In women who had C/S, the risk of non-EBF three days following birth was 1.468-fold higher (95% CI, 1.233–1.748) after adjusting for maternal educational level and residential region.

According to secondary analysis of the WHO Global Survey [27] using data from several countries, the adjusted OR for EIBF was 0.28 (95% CI: 0.22–0.37; *P* <  0.001) for women who had C/S, indicating an evidently high risk of non-EIBF in cases of C/S. Prior et al. [9] also observed that the EIBF rate in cases of C/S was low; their calculated pooled OR was 0.57 (95% CI: 0.50–0.64; *P* <  0.00001). Regan et al. [28] reported that women with successful VD were 1.42-fold more likely to have EIBF than women who had a planned C/S after a previous C/S (95% CI: 1.30–1.56) and that those who had C/S after an unsuccessful VD attempt were 1.15-fold more likely to have EIBF than women who had a planned C/S after a previous C/S (95% CI: 1.01–1.31).

The results of the present study should be considered in the context of some limitations. As the data were obtained solely from the 2013 TDHS, factors associated with breastfeeding not included in the survey were not analysed. As such, it is possible that mode of delivery and breastfeeding are associated with the characteristics of the hospitals (such as type, region, and size) where women give birth; however, the 2013 TDHS data were not sufficient to evaluate this possibility. In addition, the data could not be used to determine if any of the women delivered babies in hospitals that were not baby-friendly. Moreover, the 2013 TDHS did not collect data about the women’s pre-delivery intentions to breastfeed. It is possible that, before delivery, some of the women had decided not to breastfeed or to engage in less than ideal breastfeeding practices, but such data were not included in the 2013 TDHS. The number of deliveries that could be considered unnecessary C/S was not known and it could not be determined if any of the women had valid barriers to breastfeeding. The survey also did not include any data concerning the number of women who had instrumental or anaesthetic VD, which can cause a delay in mother-baby contact.

Despite these limitations, the present study has some strengths that should be acknowledged. The study was based on a subsample of a nationally representative survey that gathered high-quality data. The retrospective cohort design facilitated a thorough examination of the relationship between C/S and breastfeeding practices. Several potential influences were excluded in the process of selecting the study sample and some other confounders were controlled for via standardisation; thus, the measurement of the effect of C/S on breastfeeding practices was refined.

## Conclusions

According to the present findings, C/S significantly increases the risk of non-EIBF and non-EBF, after controlling for mothers’ sociodemographic and reproductive characteristics. This indicates that unnecessary C/S negatively affects not only maternal health but also neonatal health. Policies that promote breastfeeding and the incorporation of mother-friendly policies in baby-friendly hospitals are essential for increasing the rates of both EIBF and EBF.

## Data Availability

The data from the 2013 TDHS can be acquired using a formal application submitted to the Hacettepe University Institute of Population Studies via their official website (http://www.hips.hacettepe.edu.tr/tnsa/download.php).

## Abbreviations

C/S: Caesarean section
CI: Confidence interval
EBF: Exclusive breastfeeding
EIBF: Early initiation of breastfeeding
OR: Odds ratio
RR: Relative risk
SIR: Standardised incidence rate
SRR: Standardised rate ratio
TDHS: Turkey Demographic and Health Survey
VD: Vaginal delivery
WHA: World Health Assembly
WHO: World Health Organization
